# Supplementary figures and images for: Role of FMRP in rapid antidepressant effects and synapse regulation
Source: Mol Psychiatry. 2021 Jan 12;26(6):2350–62. doi: 10.1038/s41380-020-00977-z (PMC8440195; doi:10.1038/s41380-020-00977-z)

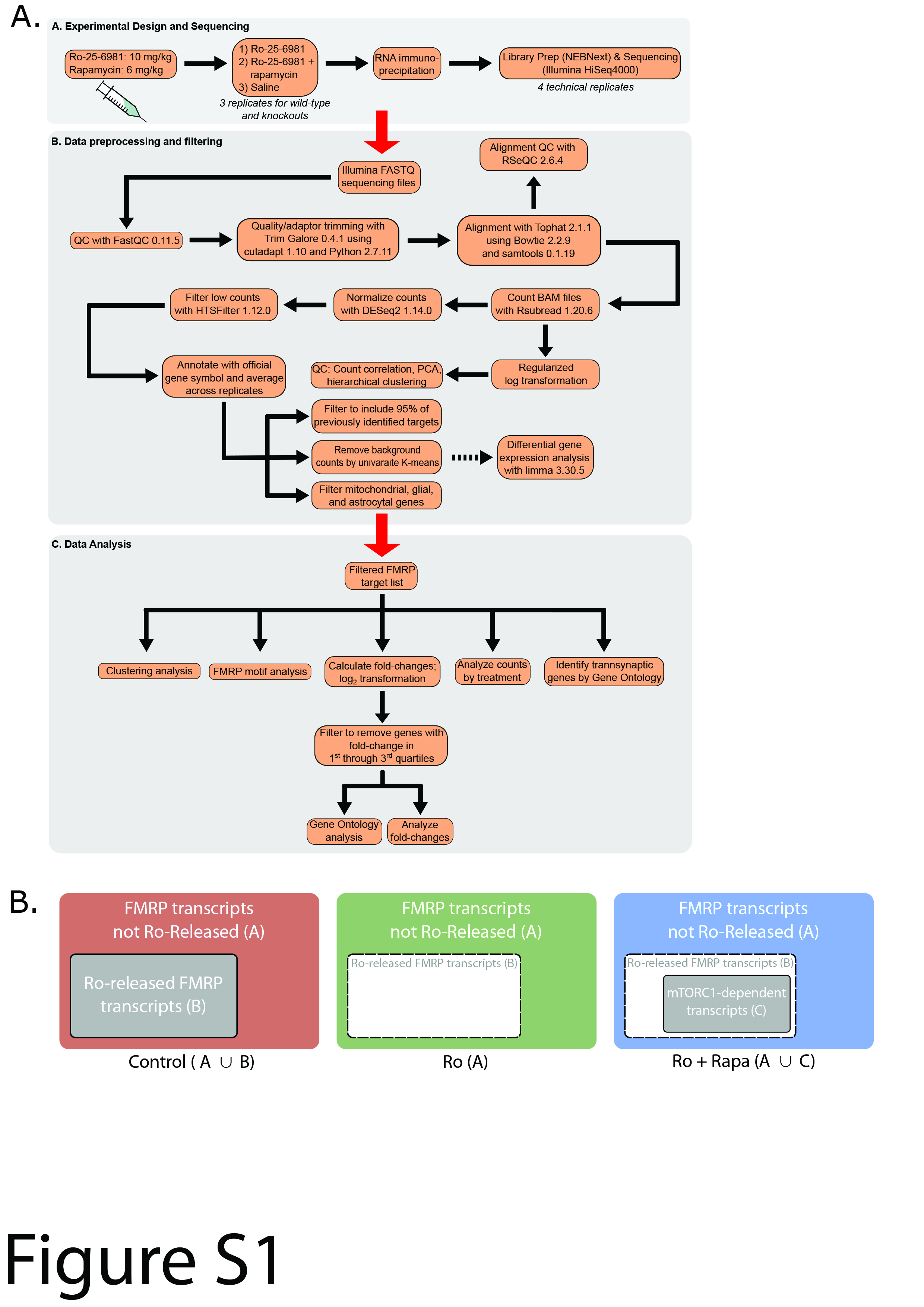

Supplement: Supplementary file 2 — Supplemental Figure 1 [file 41380_2020_977_MOESM2_ESM.tif]

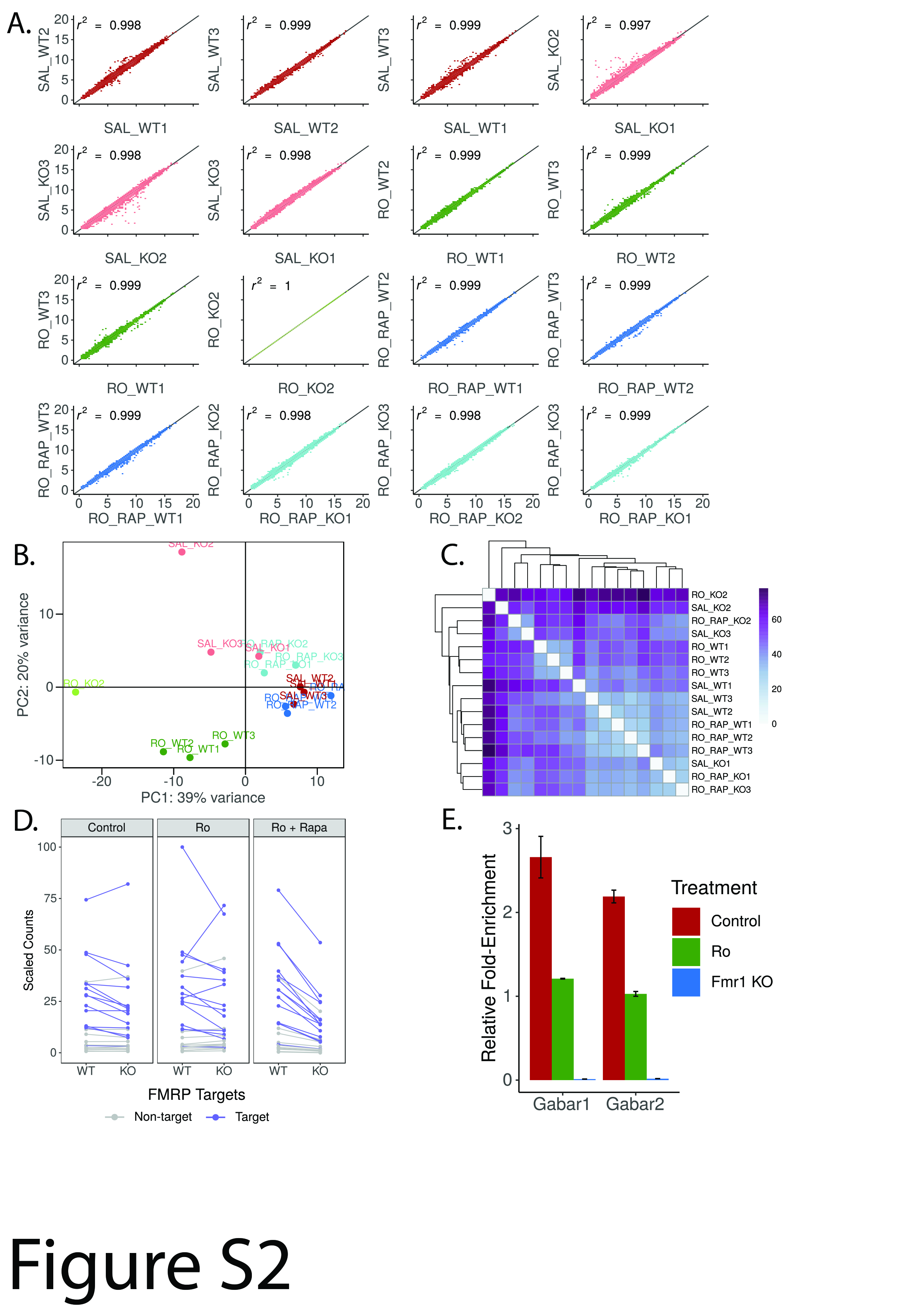

Supplement: Supplementary file 3 — Supplemental Figure 2 [file 41380_2020_977_MOESM3_ESM.tif]

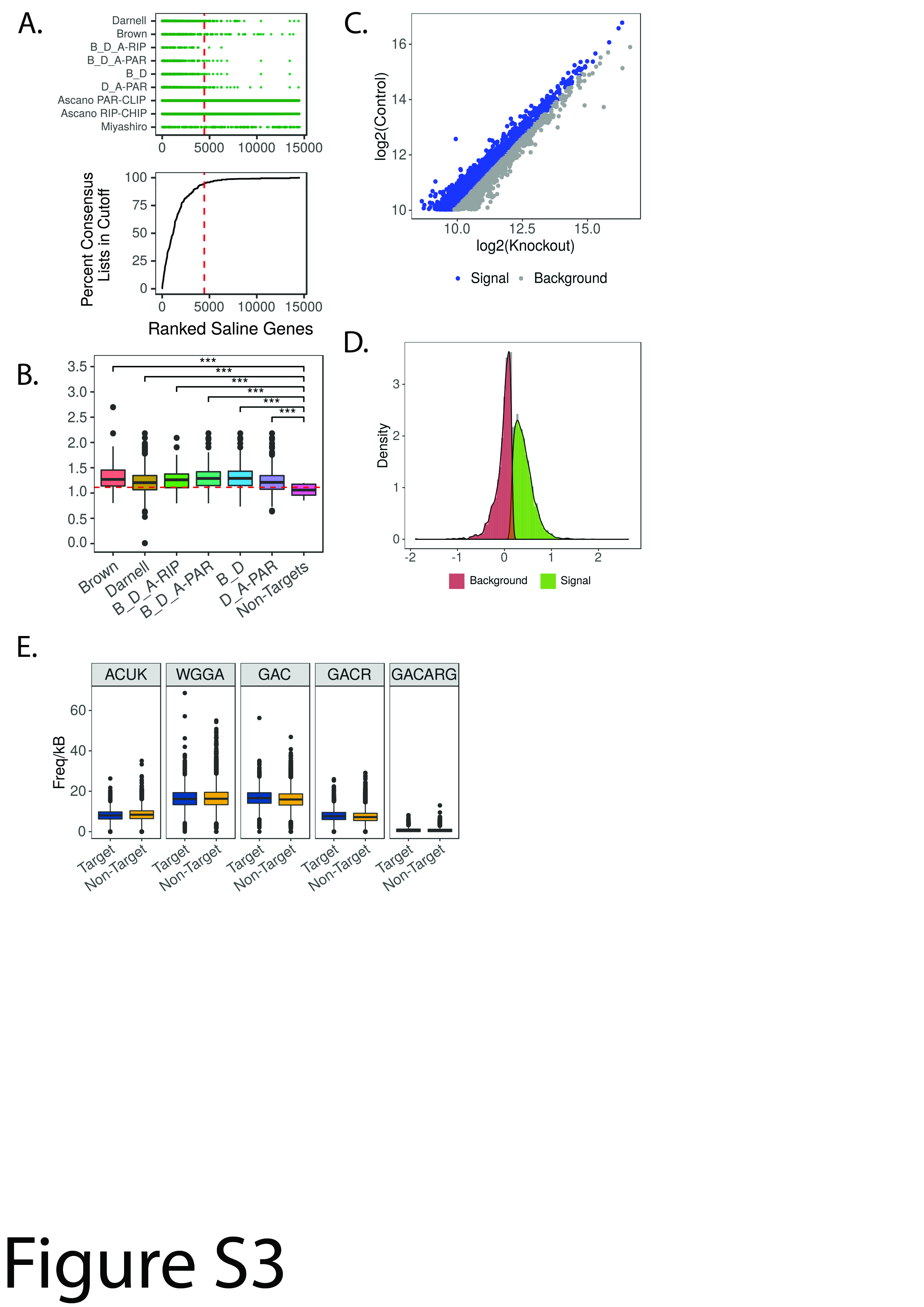

Supplement: Supplementary file 4 — Supplemental Figure 3 [file 41380_2020_977_MOESM4_ESM.tif]

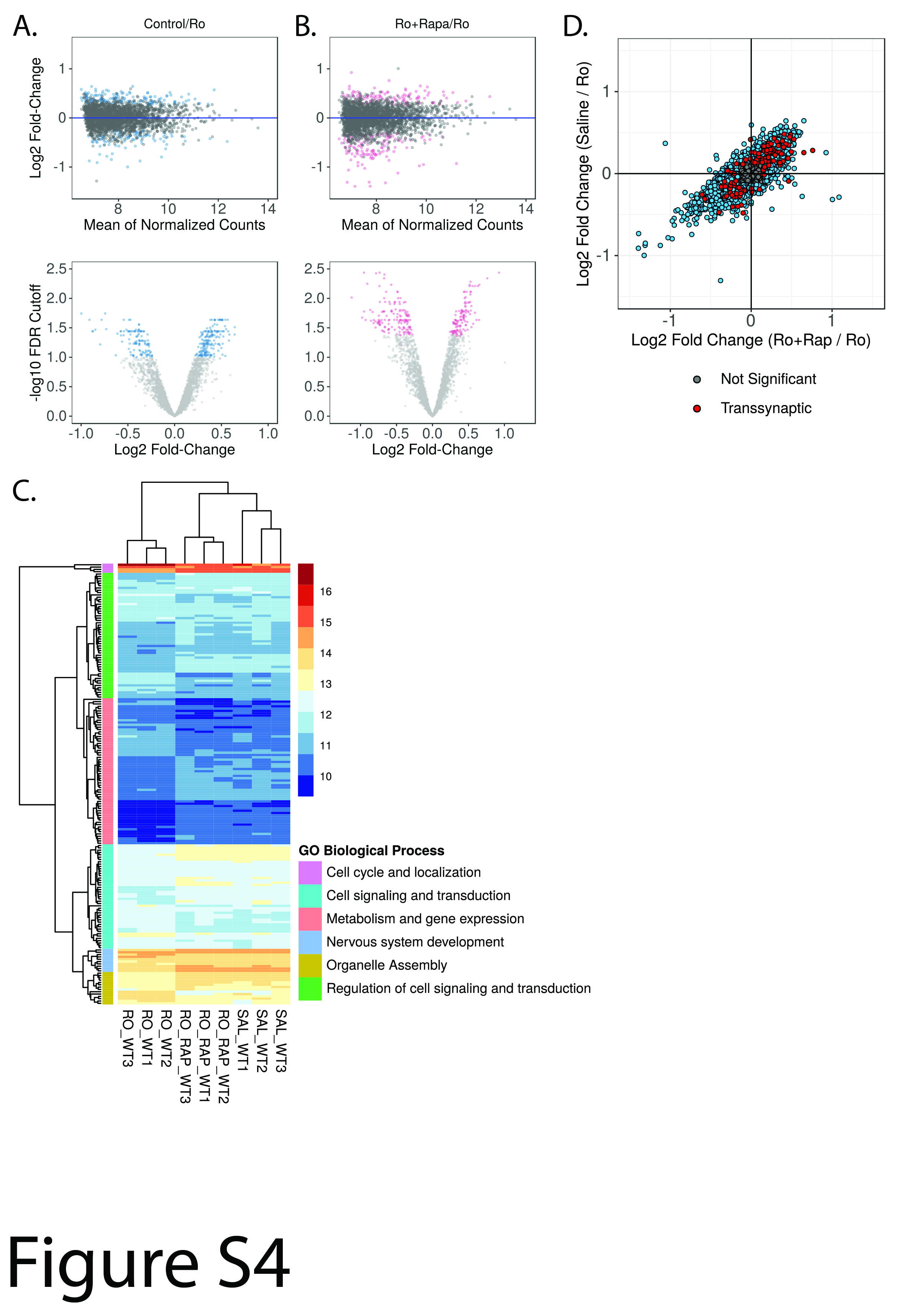

Supplement: Supplementary file 5 — Supplemental Figure 4 [file 41380_2020_977_MOESM5_ESM.tif]
